# Pharmacogenomics knowledge, attitudes, and perceptions among pharmacists in the Asir Region, Saudi Arabia: a cross-sectional analysis

**DOI:** 10.1007/s44446-025-00061-z

**Published:** 2026-02-09

**Authors:** Saud Alqahtani, Taha Alqahtani, Krishnaraju Venkatesan, Vigneshwaran Easwaran, Kousalya Prabahar, Premalatha Paulsamy, Rehab Ahmed, Nizar Sirag, Durgaramani Sivadasan, Hassabelrasoul Elfadil, Nouf M. Alharthi

**Affiliations:** 1 Department of Pharmacology, College of Pharmacy, King Khalid University, Abha, Asir Saudi Arabia; 2 Department of Clinical Pharmacy, College of Pharmacy, King Khalid University, Abha, Asir Saudi Arabia; 3 Department of Pharmacy Practice, Faculty of Pharmacy, University of Tabuk, 71491 Tabuk, Saudi Arabia; 4 College of Nursing, Mahalah Branch for Girls, King Khalid University, 62521 Abha, Saudi Arabia; 5 Division of Microbiology, Immunology and Biotechnology, Department of Natural Products and Alternative Medicine, Faculty of Pharmacy, University of Tabuk, 71491 Tabuk, Saudi Arabia; 6 Department of Natural Products and Alternative Medicine, Faculty of Pharmacy, University of Tabuk, 71491 Tabuk, Saudi Arabia; 7 Department of Pharmaceutics, College of Pharmacy, Jazan University, Jazan, Saudi Arabia; 8 Department of Pharmacy, King Khalid University Medical City, Asir Region, Saudi Arabia

**Keywords:** PGx, Knowledge, Attitude, Perception, Pharmacists, Saudi Arabia, Personalised medicine

## Abstract

As personalized medicine progresses in Saudi Arabia, pharmacists endure a central role in pharmacogenomics (PGx) implementation. This cross-sectional study weighed the knowledge, attitudes, and perceptions (KAP) of pharmacists in the Asir region regarding PGx, a region with limited published data. A total of 209 pharmacists took part, the majority of whom were male (68.4%) and aged 18–39 years (96.2%). Results revealed that 71.3% of pharmacists displayed poor knowledge, whereas positive attitudes (90.9%) and perceptions (87.6%) were widely expressed. Significant associations developed between knowledge and attitude (*p* = 0.015) and between knowledge and perception (*p* = 0.036). A moderate correlation was also reflected between attitude and perception (*p* < 0.001, Cramér’s V = 0.44). Logistic regression showed that pharmacists lacking previous PGx training (OR = 0.257, *p* < 0.001) or lacking experience with genetic conditions (OR = 0.389, *p* = 0.022) were significantly less likely to demonstrate good knowledge. Regardless of evident knowledge gaps, pharmacists showed a strong willingness to integrate PGx into clinical practice. These findings highlight an imperative need for structured PGx training programs, integration of PGx into pharmacy curricula, ongoing professional development workshops, and institutional strategies to support operative PGx adoption in healthcare settings across the Asir region and Saudi Arabia.

## Introduction

Pharmacogenomics (PGx), the study of how genetic variations affect individual responses to medications, is transforming the landscape of modern healthcare. PGx can optimize therapeutic outcomes and minimise adverse drug reactions by enabling personalized drug therapy tailored to a patient's genetic profile (Just et al. 2017; Elewa et al. 2015). As the healthcare system advances toward precision medicine, pharmacists are poised to play a critical role in integrating PGx into routine clinical practice (Wondrasek et al. 2024). This necessitates an understanding of their knowledge, attitudes, and perceptions (KAP) towards PGx, as well as an evaluation of the preparedness of future pharmacists currently in training (Karas Kuželički et al. 2019a).

Pharmacists' unique position in the healthcare system as medication experts underscore their importance in implementing PGx (Kim et al. 2020). They are responsible for interpreting pharmacogenomic test results, counselling patients, and collaborating with other healthcare professionals to ensure the safe and effective use of medications (Whirl-Carrillo et al. 2012). Understanding the KAP of these groups is critical for identifying knowledge gaps and designing effective educational and training interventions (Arafah et al. 2022; Khattab et al. 2023).

Knowledge encompasses the extent to which pharmacists understand fundamental concepts in PGx, including genetic variations, their impact on drug efficacy and safety, and the clinical application of genetic testing (Shah et al. 2022). Attitudes reflect their willingness and enthusiasm to embrace PGx in practice, while perceptions provide insights into how they view its relevance and feasibility in the healthcare system (Muzoriana et al. 2017; Jairoun et al. 2024). Together, these dimensions shape the readiness of pharmacy professionals to engage with PGx and its potential to revolutionize patient care.

While global interest in PGx has grown significantly, the level of awareness, educational integration, and clinical application varies widely across regions. Studies from countries such as the United Arab Emirates, Qatar, and Jordan have reported moderate to high interest among pharmacists but have also identified persistent knowledge gaps and limited implementation in practice due to insufficient training and infrastructural support (Elewa et al. 2015; Khattab et al. 2023; Jairoun et al. 2024). For instance, a national survey in the UAE revealed that although pharmacists recognize the importance of PGx, few have received formal education or hands-on experience in its application (Jairoun et al. 2024). Similarly, in Qatar, healthcare professionals acknowledged the utility of PGx but expressed concerns about training and institutional readiness (Elewa et al. 2015).

Internationally, comparable trends have been observed in Europe and Southeast Asia, where pharmacy education programs are increasingly incorporating PGx, yet real-world clinical implementation remains limited (Just et al. 2017; Siamoglou et al. 2021). Despite this growing body of literature, there is a notable lack of region-specific data from the southwestern part of Saudi Arabia, particularly the Asir region. This gap highlights the significance of the current study in situating local findings within the broader Middle Eastern and global context, as well as in identifying region-specific needs for advancing PGx in pharmacy education and practice.

Although these international and regional efforts demonstrate increasing attention to PGx, it remains uncertain how these advances are reflected at the local level, particularly in less-studied regions. Despite global advancements, the integration of PGx in pharmacy education and practice in many regions, including the Asir region, remains an area that requires further exploration and development. Further, published research reports on exploring the KAP of pharmacists in this region are also scarce. Therefore, this study aimed to understand and analyze the KAP toward PGx. Including pharmacists offers a dual perspective, providing insights into real-world application challenges and reflecting the current state of academic preparation (Siamoglou et al. 2021; Kekulandara and Wickramarachchi 2024). This dual approach ensures a more holistic understanding of the factors influencing the integration of PGx into pharmacy practice.

Further, the findings of this study hold significant implications for pharmacy education and professional development in the Asir region and beyond. Identifying knowledge deficits among pharmacists can inform curriculum enhancements, ensuring PGx is adequately covered in pharmacy programs. Furthermore, understanding attitudes and perceptions can guide the design of targeted interventions to address resistance or misconceptions about the field. Moreover, the study aimed to identify educational gaps and barriers to implementation by evaluating participants familiarity with pharmacogenomic principles, their views on the clinical relevance of these principles, and their readiness to adopt them in practice. As PGx becomes integral to modern medicine, equipping pharmacists with the necessary knowledge, skills, and attitudes is imperative. This study offers valuable insights into the current state of KAP in the Asir region, providing a foundation for efforts to integrate PGx into pharmacy education and practice, ultimately enhancing patient outcomes through personalized medicine.

## Materials and methods

### Study design and participants

A prospective, web-based, cross-sectional study was conducted among 209 pharmacists practicing in the Asir region. The study included all pharmacists willing to participate who agreed to the electronic consent provided at the start of the questionnaire. Student pharmacists, pharmacy technicians, and pharmacy assistants were excluded from the current study. The study was conducted from January to April 2023, spanning four months.

### Sample size and sampling technique

The sample size was estimated based on the reported number of registered community pharmacists in the Asir region (747) (Almaghaslah 2022). The sample size was estimated using an online sample size calculator provided by openepi.com. With a 95% confidence level and an anticipated response frequency of 50%, the calculated required sample size was 254. The non-probability convenience sampling system was used to conduct the survey. With the achieved sample size of 209 participants, the margin of error was approximately ± 5.76% (± 5.00% for n = 254) at a 95% confidence level, which remains within acceptable limits for cross-sectional survey studies, although the precision is slightly reduced. This shortfall of sample size was mostly due to the practical limitations typical of survey research, such as lower-than-expected response rate and reliance on non-probability convenience sampling, compounded by scheduling and workload pressures among pharmacists during the study window.

### Questionnaire

The self-administered survey questionnaire was developed based on previously published literature (15–18). It was organized into four distinct sections. The first section gathered socio-demographic data. The second section comprises 15 knowledge-related fixed-response questions, the third section contains 10 items related to attitudes, and the last section consists of 17 items evaluating perceptions toward genomic medicine and PGx. Both the attitude and perception domains consist of three-point Likert scale questions. Among the knowledge questions, Q. Nos. 2, 5, 9–15 were basic knowledge questions, and the remaining questions pertained to the applied knowledge of PGx. The questionnaire was originally prepared in English and reviewed by an expert panel to ensure face validity. The reliability of the questionnaire was ensured by estimating the internal consistency using a pilot study with a sample of 20 pharmacy academicians. The estimated Cronbach's alpha was found to be 0.62. Although the Cronbach’s alpha of 0.62 is slightly below the commonly accepted threshold of 0.70, it is considered acceptable for exploratory research, particularly in studies measuring multidimensional constructs such as knowledge, attitude, and perception.

### Scoring system

The knowledge scores were calculated based on the correct responses provided by the study participants. Each correct answer was scored 1, and each incorrect answer was scored 0. The scores of each item were summed up to obtain the total score. The maximum points expected for each participant were 15, and the minimum was zero. After summing up the score, it was converted to a 0 to 100% scale. Then, it was categorized into two categories: ≤ 60% (Poor knowledge – fewer than 9 points) and above 60% (Good knowledge – 9 points or more). The 3-point Likert scale assessed attitude and perception, with scores ranging from 3 to 1. Agree = 3, Neutral = 2 and Disagree = 1. The scores for each item were summed up to obtain the total score, and then it was categorized as either positive or negative. The midpoint between the scores was decided as the cut-off point for categorization. The maximum possible score for attitude is 30, and the minimum is 10, where a score of ≤ 20 is considered negative and a score of > 20 is considered positive. Similarly, the maximum possible score for the perception domain is 51, and the minimum is 17. The scores of ≤ 34 and > 34 were considered negative and positive perceptions, respectively.

### Ethical considerations

The study protocol was approved by the Research Ethics Committee at King Khalid University (ECM#2023–505, dated 12^th^ January 2023). Participation in the research is voluntary. The purpose and protocol were detailed to all the respondents, and electronic consent was obtained. The confidentiality of the study participants was maintained. All the data were analyzed anonymously and in an aggregate fashion to preserve data privacy and confidentiality.

### Data collection

The survey questionnaire was uploaded to Google Forms, and a Google web link was made available through social media platforms, including Twitter, WhatsApp, and Facebook. The questionnaire included an item related to the region, which helped us separate and sort the data specifically for the southern region of Saudi Arabia. After outlining the study objectives, the research assistants shared the Google link for the questionnaire on social media. Every study participant was questioned about their consent. The findings were presented anonymously, and pharmacists were informed that all the information they provided would be kept confidential. Questions with contact information or names that could identify the patients personally were avoided.

### Data analysis

Data were analyzed using the Statistical Package for the Social Sciences (SPSS) version 23 (IBM Corp., Armonk, NY, USA). Descriptive statistics (frequencies, percentages, means, and standard deviations) were calculated for demographic variables. Associations between categorical variables were examined using the Chi-square test, and the strength of association was reported with Cramér's V. Binary logistic regression was applied to identify predictors of KAP, with odds ratios (OR) and 95% confidence intervals (CI) reported. Before logistic regression, multicollinearity was assessed, and no significant violations were observed. A *p-*value < 0.05 was considered statistically significant.

## Result

### Demographics

Table 1 demonstrates the demographic and professional characteristics of pharmacists. A total of 209 pharmacists completed the survey, comprising 143 males (68.4%) and 66 females (31.6%). Most pharmacists (96.2%) were aged between 18 and 39 years, while a smaller proportion (3.8%) were in the 40 to 59 age group. The educational background of pharmacists varied, with 95.2% holding a bachelor’s degree, 3.3% possessing a master’s degree, and 1.4% holding a Ph.D. In terms of professional roles, 87 pharmacists (41.6%) worked in sales, 61 (29.2%) in academia, 53 (25.4%) in hospitals, 4 (1.9%) in industry, and another 4 (1.9%) in Regulatory institutions. Regarding work experience, the greater part (66%) had < 5 years of experience, followed by 34% with > 5 years. Notably, 86.1% of pharmacists reported no prior exposure to genetic issues, while 13.9% had previous exposure. Additionally, 26.8% of participants had received training or education in PGx, while 73.2% had not. These insights reflect the diverse demographic and professional backgrounds of the pharmacists.

**Table 1:** Demographic and professional characteristics of pharmacists

Characteristics	Frequency n (%)
**Gender**	
Male	143 (68.4)
Female	66 (31.6)
**Age group**	
18–39 years	201 (96.2)
40–59 years	8 (3.8)
**Educational Qualification**	
Bachelor’s Degree	199 (95.21)
Masters	7 (3.35)
PhD	3 (1.44)
**Practice area**	
Academia	61 (29.2)
Sales	87 (41.6)
Hospital	53 (25.4)
Industry	4 (1.9)
Regulatory	4 (1.9)
**Experience in years**	
≤ 5 years	138 (66.0)
> 5 years	71 (34.0)
**Exposure to a genetic problem**	
No	180 (86.1)
Yes	29 (13.9)
**Training on PGx**	
No	153 (73.2)
Yes	56 (26.8)

### Frequency distribution of KAP

**Fig. 1:**
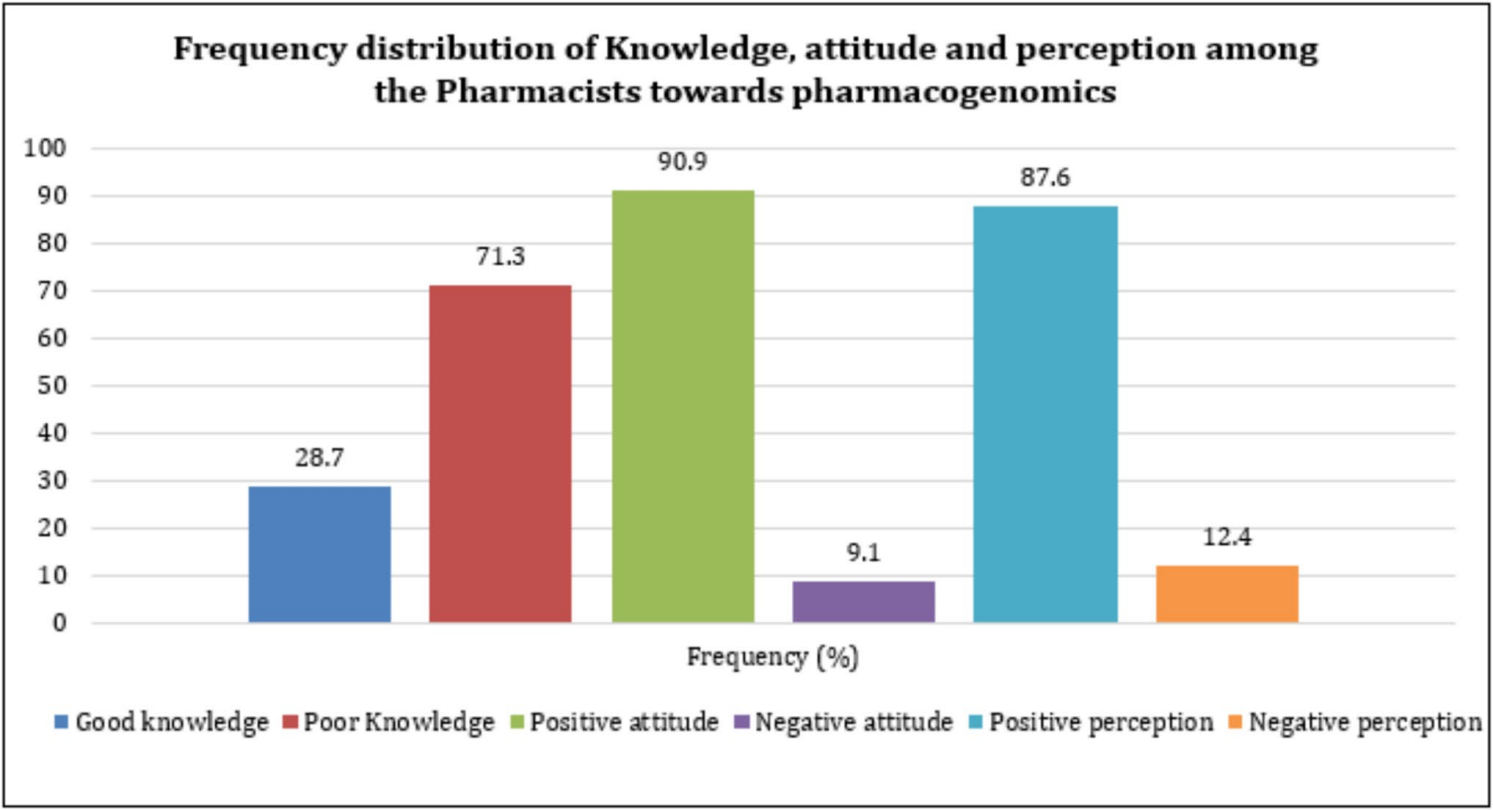
Frequency distribution of KAP among the pharmacists towards PGx

According to Fig. 1, although most participants have a positive attitude (90.9%) and a positive perception (87.6%), the majority still lack adequate knowledge (71.3%).

### Association between the domains

Chi-square and Cramér's V tests were conducted to assess the relationship and its strength between knowledge, attitude, and perception. There is a statistically significant association between knowledge and attitude (*p* = 0.015**) as well as perception (*p* = 0.036*), indicating that individuals with better knowledge are more likely to have a positive attitude and perception. There is a strong relationship between attitude and perception (*p* = 0.000), which suggests that a positive attitude is strongly linked to a positive perception, indicating that perception may be influenced by attitude (Table 2).

**Table 2:** Association between the domains

Domains	Chi-Square	*‘p’* value	Cramér’s ‘V’
Knowledge Vs Attitude	5.938	0.015*	0.17 (Weak Association)
Knowledge Vs Perceptions	4.411	0.036*	0.15 (Weak Association)
Attitude Vs Perceptions	39.643	0.000*	0.44 (Moderate Association)

*p < 0.05 -significant

### Pharmacists’ knowledge of PGx

The knowledge of the participants was assessed in two aspects: basic knowledge and applied knowledge based on practical experience. Among the knowledge questions, Q. Nos. Nos. 2, 5, 9–15 were basic knowledge questions, and the remaining questions were based on applied knowledge, drawing on practical experience. The survey results from Table 3 reveal that pharmacists possess a satisfactory level of applied knowledge based on practice, with a strong awareness of certain key concepts, which is significantly associated with participants who had training exposure to PGx (*p* < 0.05). In addition, although it is not statistically significant, pharmacists with more years of experience demonstrate a higher level of applied knowledge in the practice of PGx. Ironically, the samples lack knowledge of the basics of PGx, irrespective of training on PGx and years of experience.

**Table 3:** Association of pharmacists’ knowledge with years of experience and training on PGx

Items and Response Distribution on Knowledge		Training on PGx			Experience (years)		
		No (153)	Yes (56)	χ2 *'p'* value	≤ 5 (138)	> 5 (71)	χ2 '*p*' value
		**%**			**%**		
K1. Subtle differences in a person's genome can have a major impact on how the person responds to medications	Wrong	24.2	10.7	0.033*	21.0	19.7	0.8
	Correct	75.8	89.3		79.0	80.3	
K2. Genetic determinants of drug response change over a person lifetime	Wrong	86.9	83.9	0.6	84.1	90.1	0.2
	Correct	13.1	16.1		15.9	9.9	
K3. Genetic variants can account for 95% of the variability in drug disposition and effects	Wrong	50.3	33.9	0.035*	48.6	40.8	0.3
	Correct	49.7	66.1		51.4	59.2	
K4. The package insert for warfarin includes a warning about altered metabolism in individuals who have specific genetic variants	Wrong	45.8	25.0	0.007*	44.9	31.0	0.1
	Correct	54.2	75.0		55.1	69.0	
K5. PGx testing is currently available for most medications	Wrong	82.4	76.1	0.2	82.6	69.0	0.0
	Correct	17.6	23.9		17.4	31.0	
K6. Genetic changes can cause adverse reactions	Wrong	20.3	10.7	0.1	20.3	12.7	0.2
	Correct	79.7	89.3		79.7	87.3	
K7. The FDA recommends PGx testing for certain drugs	Wrong	34.0	16.1	0.014*	30.4	26.8	0.6
	Correct	66.0	82.1		69.6	71.8	
K8. Genes can be activated or deactivated by other genes	Wrong	47.7	26.8	0.007*	40.6	45.1	0.5
	Correct	52.3	73.2		59.4	54.9	
K9. Environmental factors, such as cigarette smoke, can affect gene activity	Wrong	31.4	19.6	0.1	29.7	25.4	0.5
	Correct	68.6	80.4		70.3	74.6	
K10. Every cell of the body contains the whole genome	Wrong	88.9	83.9	0.3	87.7	87.3	0.9
	Correct	11.1	16.1		12.3	12.7	
K11. Healthy parents can have a child with a hereditary disease	Wrong	24.8	23.2	0.8	30.4	12.7	0.0
	Correct	75.2	76.8		69.6	87.3	
K12. The onset of certain diseases is influenced by a combination of genetic, environmental, and lifestyle factors	Wrong	13.1	21.4	0.1	16.7	12.7	0.4
	Correct	86.9	78.6		83.3	87.3	
K13. All serious diseases are hereditary	Wrong	89.5	85.0	0.8	86.2	84.5	0.7
	Correct	10.5	15.0		13.8	15.5	
K14. Adenine (A) only pairs with cytosine (C), and Thymine (T) only pairs with Guanine	Wrong	56.2	60.7	0.6	50.7	70.4	0.0
	Correct	43.8	39.3		49.3	29.6	
K15. Humans have 48 chromosomes	Wrong	56.9	48.2	0.3	55.1	53.5	0.8
	Correct	43.1	51.8		44.9	46.5	

*p < 0.05 -significant, χ2 -Chi -Square

### Pharmacists attitude toward PGx

The survey results highlight pharmacists’ strong positive attitude towards PGx, irrespective of their training on PGx and their years of experience. Although there was no significant difference, the attitude of the samples was positive in all key concepts tested in this survey, except for the donation of genetic materials to the biobank. This statement, to some extent, relates to the basic knowledge of PGx; however, the samples lacked adequate knowledge on the same, which negatively reflected in their attitude (Table 4).

**Table 4:** Association of pharmacists’ attitude with years of experience and training on PGx

Items and Response Distribution on Attitude		Training on PGx			Experience (years)		
		No	Yes	χ2 *'p'* value	≤ 5	> 5	χ2 '*p*' value
		**%**			**%**		
A1. PGx testing will help decrease the number of adverse drug reactions	Disagree	5.88	10.7	0.443	6.52	8.45	0.744
	Neutral	15.7	12.5		15.9	12.7	
	Agree	78.4	76.8		77.5	78.9	
A2. PGx testing will help decrease the cost of developing new drugs	Disagree	7.84	14.3	0.128	8.7	11.3	0.097
	Neutral	27.5	16.1		29	15.5	
	Agree	64.7	69.6		62.3	73.2	
A3. PGx testing will reduce the time required to determine the optimal dose for patients taking warfarin	Disagree	6.54	8.93	0.475	7.25	7.04	0.057*
	Neutral	27.5	19.6		30.4	15.5	
	Agree	66	71.4		62.3	77.5	
A4. PGx testing will help reduce ADR due to warfarin	Disagree	6.54	1.79	0.066	5.07	5.63	0.852
	Neutral	29.4	17.9		27.5	23.9	
	Agree	64.1	80.4		67.4	70.4	
A5. At some point in my life, I might consider having a genetic test to find out my risk of developing various genetic diseases	Disagree	17	17.9	0.793	15.2	21.1	0.325
	Neutral	18.3	14.3		19.6	12.7	
	Agree	64.7	67.9		65.2	66.2	
A6. If I were diagnosed with cancer, I would consider having my genes analysed to choose a cancer treatment with fewer side effects	Disagree	8.5	7.14	0.154	8.7	7.04	0.845
	Neutral	13.7	25		17.4	15.5	
	Agree	77.8	67.9		73.9	77.5	
A7. If I had a family history of diabetes, I would consider having my genes analysed to help me make lifestyle choices and decisions about interventions that may prevent diabetes from developing	Disagree	5.88	5.36	0.983	5.8	5.63	0.483
	Neutral	20.3	19.6		22.5	15.5	
	Agree	73.9	75		71.7	78.9	
A8. I would like to donate my genetic materials to the Biobank	Disagree	35.3	35.7	0.241	33.3	39.4	0.276
	Neutral	34	23.2		34.8	23.9	
	Agree	30.7	41.1		31.9	36.6	
A9. I would be interested in attending a PGx course and/or an education seminar	Disagree	7.84	7.14	0.36	7.25	8.45	0.28
	Neutral	20.9	12.5		21.7	12.7	
	Agree	71.2	80.4		71	78.9	
A10. I would like to participate in genetic research	Disagree	19	7.14	0.078	10.9	25.4	0.22
	Neutral	22.2	19.6		21.7	21.1	
	Agree	58.8	73.2		67.4	53.5	

*p < 0.05 -significant; χ2 -Chi -Square

### Pharmacists perceptions of PGx

The survey results in Table highlight pharmacists’ positive perception of PGx as a crucial field within pharmacy practice and education. The concepts "PGx was taught in Pharmacy School" and "I know PGx tests are used in Saudi Arabia" showed a significant association with the years of experience of the samples and their exposure to training on PGx (*p* < 0.05).

Table 5 presents the association between pharmacists’ perceptions of PGx and their years of experience and training in the subject. The data show a significant relationship between PGx education in pharmacy school and both training and years of experience, with those having over five years of experience or prior training being more likely to agree that PGx was taught. Similarly, the importance of PGx in pharmacy and the necessity for pharmacists to understand it were generally acknowledged, regardless of training or experience. However, a notable percentage of pharmacists expressed uncertainty about the reliability of PGx information sources and their implementation in Saudi Arabia. Additionally, while the majority agreed on the need for PGx in pharmacy curricula and continuing education, a smaller proportion felt confident in their ability to consult on therapy changes following PGx testing. Despite these variations, most respondents believed that PGx should play a role in future pharmacy practice, indicating an overall positive perception of its importance and future integration into healthcare.

**Table 5:** Association of pharmacists perception with years of experience and training on PGx

Items and Response Distribution on Perception		Training on PGx			Experience (Years)		
		No	Yes	χ2 '*p*'value	≤ 5	> 5	χ2 '*p*-value
		**%**			**%**		
P1. PGx was taught in Pharmacy School	Disagree	14.8	14.3	0.000*	1.4	12.7	0.002*
	Neutral	26.1	25.0		16.7	12.7	
	Agree	62.0	60.7		81.9	74.6	
P2. PGx is an important field in Pharmacy	Disagree	4.6	7.1	0.737	36.2	31.0	0.356
	Neutral	15.0	16.1		27.5	22.5	
	Agree	80.4	76.8		36.2	46.5	
P3. Pharmacists must know PGx	Disagree	5.9	7.1	0.643	4.3	9.9	0.279
	Neutral	15.7	10.7		15.2	12.7	
	Agree	78.4	82.1		80.4	77.5	
P4. PGx should be added to the curriculum in Pharmacy School	Disagree	6.5	7.1	0.864	6.5	7.0	0.886
	Neutral	15.0	17.9		16.7	14.1	
	Agree	78.4	75.0		76.8	78.9	
P5. PGx should be taught in Pharmacy Continuing Education Seminars	Disagree	7.2	5.4	0.83	7.2	5.6	0.615
	Neutral	17.0	19.6		15.9	21.1	
	Agree	75.8	75.0		76.8	73.2	
P6. If adequately trained in PGx, I should be able to provide consultation on its clinical use	Disagree	5.9	10.7	0.383	6.5	8.5	0.252
	Neutral	13.1	16.1		16.7	8.5	
	Agree	81.0	73.2		76.8	83.1	
P7. If trained, I would be confident in advising on therapy modifications based on PGx test results	Disagree	5.2	8.9	0.494	5.8	7.0	0.571
	Neutral	21.6	25.0		24.6	18.3	
	Agree	73.2	66.1		69.6	74.6	
P8. Training will help Pharmacists identify medicines requiring PGx testing	Disagree	5.2	8.9	0.332	5.8	7.0	0.654
	Neutral	17.6	10.7		17.4	12.7	
	Agree	77.1	80.4		76.8	80.3	
P9. I know reliable sources of info on PGx	Disagree	46.4	37.5	0.169	44.2	43.7	0.332
	Neutral	26.1	21.4		27.5	19.7	
	Agree	27.5	31.1		28.3	36.6	
P10. I know PGx tests are used in Saudi Arabia	Disagree	55.6	52.9	0.051*	58.7	39.4	0.03*
	Neutral	22.9	23.2		20.3	28.2	
	Agree	21.6	33.9		21.0	32.4	
P11. The PGx test will control medicine costs	Disagree	7.8	10.7	0.784	8.0	9.9	0.332
	Neutral	24.2	25.0		27.5	18.3	
	Agree	68.0	64.3		64.5	71.8	
P12. PGx is relevant to my current practice	Disagree	38.6	28.6	0.319	35.5	36.6	0.155
	Neutral	22.2	30.4		28.3	16.9	
	Agree	39.2	41.1		36.2	46.5	
P13. Pharmacists must recommend PGx in their clinical practice	Disagree	9.8	7.1	0.712	9.4	8.5	0.905
	Neutral	19.0	16.1		18.8	16.9	
	Agree	71.2	76.8		71.7	74.6	
P14. In the future, health providers must consult Pharmacists on PGx testing	Disagree	5.9	5.4	0.716	5.1	7.0	0.814
	Neutral	20.9	16.1		20.3	18.3	
	Agree	73.2	78.6		74.6	74.6	
P15. In the future, providers must consult Pharmacists on therapy changes after PGx testing	Disagree	7.8	5.4	0.765	7.2	7.0	0.939
	Neutral	21.6	19.6		21.7	19.7	
	Agree	70.6	75.0		71.0	73.2	
P16. In the future, Pharmacists should use PGx tests for medication therapy management	Disagree	8.5	5.4	0.708	8.0	7.0	0.742
	Neutral	22.9	21.4		23.9	19.7	
	Agree	68.6	73.2		68.1	73.2	
P17. In the future, pharmacists should be responsible for conducting PGx testing as part of routine clinical practice	Disagree	9.2	8.9	0.492	8.7	9.9	0.962
	Neutral	33.3	25.0		31.2	31.0	
	Agree	57.5	66.1		60.1	59.2	

*p < 0.05 -significant; χ2 -Chi -Square

### Multiple logistic regression analysis on KAP towards

Based on the demographic variables, regarding gender, males have an OR = 0.896 (95% CI: 0.497–1.615, *p* = 0.715), in knowledge, meaning they have slightly lower odds of good knowledge compared to females, but this is not statistically significant. Males have an OR = 0.107 (95% CI: 0.014–0.818, *p* = 0.031), indicating they are significantly less likely to have a positive attitude compared to females (significant because *p* < 0.05). There was no significant relationship found between perception and gender. In the age group, younger individuals have an OR = 0.096 (95% CI: 0.012–0.799, *p* = 0.030), indicating they are significantly less likely to possess sound knowledge compared to middle-aged individuals (*p* < 0.05). However, this difference had no significant effect on attitude and perception.

The educational qualification, practice area, and years of experience had no significant effect on knowledge, attitude, or perception. Pharmacists who have not been exposed to a genetic problem have an OR of 0.389 (95% CI: 0.173–0.872, *p* = 0.022), indicating they are significantly less likely to have good knowledge compared to those who have been exposed (*p* < 0.05). Similarly, those without PGx training have an OR = 0.257 (95% CI: 0.134–0.490, *p* < 0.001), meaning they are significantly less likely to have good knowledge compared to those with training (highly significant, *p* < 0.001). These two variables did not show any significant relationship with attitude and perception (Table 6).

**Table 6:** Bivariate Logistic regression for KAP

	Knowledge (Good and Poor)		Attitude (Positive & negative)		Perception (Positive & negative)	
	OR (95% CI)	*‘p*’	OR (95% CI)	*‘p*’	OR (95% CI)	*‘p*’
**Sex**						
Male	0.896(0.497–1.615)	0.715	0.107 (0.014–0.82.014.82)	0.031*	0.476 (0.171–1.324	0.16
Female	Reference					
**Age group**						
Young	0.096 (0.012–0.799.012.799)	0.030*	0.000 (0.000)	0.999	0.000 (0.000)	0.999
Middle age	Reference					
**Educational Qualification**						
Bachelors	0.000 (0.000)	0.999	0.000 (0.000)	0.999	0.000 (0.000)	0.999
Masters	0.000 (0.000)	0.999	1.00 (0.000)	1.00	0.000 (0.000)	0.999
PhD	Reference					
**Practice area**						
Academia	0.231(0.023–2.36)	0.216	0.000 (0.000)	0.999	4.750 (0.39–56.71)	0.218
Sales	0.235(0.024–2.35)	0.218	0.000 (0.000)	0.999	1.738 (0.17–17.94)	0.643
Hospital	0.256 (0.025–2.62)	0.251	0.000 (0.000)	0.999	2.611 (0.23–29.29)	0.436
Industry	0.111 (0.005–2.73)	0.178	1.000 (0.000)	1.000	1.000 (0.04–24.55)	1.00
Regulatory	Reference					
**Experience in years**						
≤ 5 years	0.641 (0.360–1.14)	0.132	0.888 (0.32–2.45)	0.817	0.847 (0.35–2.06)	0.713
> 5 years	Reference					
**Exposure to a genetic problem**						
No	0.389 (0.173–0.872.173.872)	0.022*	0.321 (04–2.51)	0.279	0.000 (0.000)	0.998
Yes	Reference					
**Training on PGx**						
No	0.257 (0.134–0.490.134.490)	0.00*	0.708 (0.23–2.23)	0.555	1.008 (0.399–2.54)	0.987
Yes	Reference					

*p < 0.05 -significant

## Discussion

The findings of this study offer a few critical insights into the demographic and professional backgrounds of pharmacists, their knowledge of PGx, and their attitudes and perceptions towards integrating it into pharmacy practice. The results indicate that while pharmacists exhibit a relatively low level of knowledge (71.3%), they demonstrate a predominantly positive attitude (90.9%) and a strong positive perception (87.6%) toward PGx. Cramér's V analysis revealed a significant association between knowledge and both attitude (*p* = 0.015) and perception (*p* = 0.036), suggesting that individuals with better knowledge tend to exhibit more positive attitudes and perceptions. However, a stronger association was observed between attitude and perception (*p* = 0.000, V = 0.44), indicating that mentality has a greater influence on perception than knowledge does.

This positive perception and attitude, despite knowledge gaps, suggests that factors such as personal experiences, cultural influences, and social desirability bias may play a more substantial role in shaping pharmacists’ attitudes than their actual knowledge of PGx. Similar findings have been reported in previous studies, where non-cognitive factors such as social influences, ethical considerations, and perceived professional roles have shaped attitudes toward emerging healthcare technologies, often independent of factual knowledge (McCullough et al. 2021). The stronger association between attitude and perception aligns with the Theory of Planned Behaviour (Alsulami 2025), which posits that attitude is a key determinant of perception and behavioural intentions.

The high percentage of pharmacists holding positive perceptions and recognizing the importance of PGx suggests strong enthusiasm for its implementation, despite notable knowledge gaps regarding its practical applications. These findings are consistent with prior studies, which have identified insufficient training and misinformation as barriers to the effective integration of PGx in pharmacy practice (Moaddeb et al. 2015). Our demographic analysis further contextualizes these insights, revealing that the majority of the 209 respondents were male (68.4%), young (96.2% aged 18–39), and highly educated, with 99.3% holding at least a bachelor degree. Participants represented a range of professional backgrounds, including sales (42.1%), academia (33.4%), and hospital settings (27.3%). Notably, while 26.8% of pharmacists had received prior training in PGx, 73.2% did not have an imbalance, which likely contributed to the considerable variability in pharmacists’ knowledge, even though attitudes and perceptions remained broadly positive.

Despite the strong recognition of the importance of PGx, significant knowledge gaps remain, particularly regarding the availability of PGx testing and its real-world applications. Interestingly, while training in PGx was significantly associated with improved applied knowledge, it did not lead to a similar improvement in basic knowledge scores. This may be due to the nature and quality of the training, which is often short-term, practice-oriented, and focused on clinical application rather than theoretical content. As such, pharmacists may gain functional competencies without acquiring a deep understanding of fundamental genomic concepts. This highlights the need for a more comprehensive educational approach that integrates both basic and applied PGx, ideally through formal undergraduate and postgraduate pharmacy curricula.

A previous study conducted in Saudi Arabia reported that only one-third (33%) of community pharmacists demonstrated good knowledge, while 66.3% had poor knowledge of PGx and genetics (Alrabiah et al. 2023). These findings are consistent with our results and with international trends, further emphasizing the widespread need for structured PGx education and training (Cicali et al. 2020). While 78.5% of pharmacists supported the inclusion of PGx in pharmacy curricula and 76.1% endorsed its integration into continuing education, the survey also identified barriers to implementation, including limited accessibility, inadequate training, and the absence of standardized clinical guidelines. A significant proportion (71.8%) of pharmacists believed they could recommend therapy adjustments after PGx training, yet a lack of institutional support and regulatory frameworks hinders the practical application of this knowledge in practice. Similar concerns have been raised in previous studies, where the absence of well-defined clinical workflows and standardized PGx guidelines has limited pharmacists' ability to effectively integrate PGx into patient care (Caudle et al. 2016).

Confidence in PGx-based decision-making was high among pharmacists, with 75.6% believing that healthcare providers should consult them on PGx testing and 72.2% supporting pharmacist-led therapy adjustments. However, a notable gap remains between perceived readiness and actual implementation capabilities, reflecting findings from earlier research, where pharmacists often reported enthusiasm for PGx but lacked structured guidelines and institutional backing to support its clinical adoption (Haidar et al. 2021; Luzum et al. 2016).

Statistical analysis of demographic variables revealed that males were significantly less likely to have a positive attitude than females (OR = 0.107, *p* = 0.031), while younger pharmacists had significantly lower odds of possessing good knowledge compared to middle-aged individuals (OR = 0.096, *p* = 0.030). Interestingly, educational qualifications, practice area, and years of experience had no significant impact on knowledge, attitude, or perception, suggesting that exposure to PGx training and genetic issues plays a more crucial role than formal academic credentials alone. Pharmacists without exposure to genetic issues (OR = 0.389, *p* = 0.022) or prior PGx training (OR = 0.257, *p* < 0.001) were significantly less likely to demonstrate good knowledge, reinforcing the necessity of targeted education and training programs.

Overall, the study highlights pharmacists positive attitudes and perceptions toward PGx, despite persistent knowledge gaps that could hinder its adoption in pharmacy practice. The findings underscore the need for comprehensive PGx education, institutional support, and standardized clinical guidelines to bridge knowledge gaps and enhance real-world applications.

Prior research has emphasized the importance of integrating PGx into pharmacy curricula, providing continuing professional development opportunities, and establishing clear regulatory frameworks to support PGx implementation in clinical settings (Caraballo et al. 2017). Moving forward, efforts should focus on expanding structured training programs, addressing misconceptions, and ensuring that pharmacists have access to reliable PGx information and practical guidelines. Institutional investment in personalized PGx education and clinical integration will be crucial in enhancing pharmacists confidence and capability to effectively incorporate PGx into patient care. Several studies have emphasized the critical need for structured PGx education within pharmacy and medical curricula, as well as the development of standardized clinical guidelines to support its implementation in patient care settings.

A global survey by Kuželički et al. revealed that while PGx education is increasingly included in pharmacy programs, it is often fragmented and inconsistently integrated, with substantial gaps in standalone or clinically applied content across institutions and countries (Karas Kuželički et al. 2019b). In clinical settings, the lack of consistent education and clinical decision support tools has been identified as a barrier to implementation, prompting global initiatives like the CPIC (Clinical Pharmacogenetics Implementation Consortium) and U-PGx (Ubiquitous PGx Project) to provide accessible, evidence-based guidelines (Adams et al. 2018). Furthermore, studies by Krebs and Milani highlight that successful implementation of PGx relies heavily on proactive, panel-based testing and integration into electronic health records—approaches that require pharmacists and healthcare professionals to be proficient in interpreting genetic results and translating them into therapeutic recommendations (Krebs and Milani 2019). Concrete examples of regulatory success include the US FDA Table of Pharmacogenomic Biomarkers in Drug Labeling, which integrates PGx considerations directly into approved drug prescribing information (Kim et al. 2021). The Dutch Pharmacogenetics Working Group (DPWG) also provides a leading example, with genotype-guided dosing recommendations embedded within national e-prescribing systems (Beunk et al. 2024).

Additionally, the U-PGx initiative in Europe demonstrates how cross-national policy frameworks can drive PGx adoption by integrating clinical decision support tools into electronic health records across multiple countries (Blagec et al. 2024). Without a strong educational foundation and standardized clinical pathways supported by such regulatory models, the potential of PGx to improve drug efficacy, reduce adverse events, and support personalized medicine will remain underutilized. Therefore, harmonized education, credentialed training, and the institutionalization of guideline-driven PGx services are critical next steps.

## Limitations

This study also has some limitations. Since a non-probability sampling method was used to collect data, selection bias may have occurred, potentially overrepresenting pharmacists with more interest or awareness of PGx. Additionally, the reliance on self-reported data introduces the risk of social desirability bias, particularly in items related to perceived competence, attitudes, and willingness to engage in personalized PGx practice. Participants may have provided responses they believed were professionally favorable rather than accurate reflections of their actual knowledge or behavior.

To mitigate these biases in future studies, we recommend using randomized or stratified sampling techniques to enhance representativeness. Additionally, combining self-report tools with objective assessment methods, such as validated knowledge quizzes, chart audits, observational checklists, or simulated case evaluations, can provide a more accurate picture of pharmacists’ actual competencies and practices. Finally, as the study was cross-sectional, causal relationships cannot be established; therefore, longitudinal research may be beneficial for observing changes over time.

Further, though the Cronbach's alpha value (0.62) is slightly below the commonly accepted threshold of 0.70, it is still considered acceptable in exploratory studies, particularly when assessing constructs such as KAP that are inherently multidimensional. Nonetheless, this lower value indicates moderate internal consistency, suggesting that future research should consider refining the instrument through a larger pilot study, additional items, or confirmatory factor analysis to improve reliability.

## Future directions

To fully integrate PGx into pharmacy practice, a comprehensive and multifaceted approach is required. Standardizing PGx education by incorporating it into pharmacy curricula and offering structured continuing education programs will ensure that pharmacists develop both foundational knowledge and practical expertise. Implementing certification programs, case-based training, and hands-on clinical exposure will further enhance pharmacists competency in interpreting genetic data and optimizing drug therapy.

The development of clear clinical guidelines is also essential, as the absence of standardized protocols limits the practical application of PGx in practice. Establishing national frameworks and decision-support tools will help pharmacists integrate genetic testing into medication therapy management and collaborate more effectively with other healthcare professionals. Additionally, improving access to PGx resources and testing facilities is crucial, as limited availability remains a significant barrier. Expanding access to genetic databases, PGx testing laboratories, and cost-effective testing models will facilitate the real-world implementation of these advancements.

Furthermore, regulatory and policy support must address challenges such as insurance coverage, reimbursement policies, and ethical considerations related to the privacy of genetic data. Policymakers should develop frameworks that protect patient rights while enabling pharmacists to utilize PGx insights effectively. By addressing these challenges, pharmacists can play a leading role in precision medicine, ultimately enhancing patient outcomes and ensuring safer, more personalized drug therapy. In addition, sustained funding from governmental bodies and research institutions is essential to support infrastructure development, testing accessibility, and training the PGx workforce. Advocacy for national policy initiatives that prioritize PGx integration can accelerate adoption at the institutional and health system levels.

## Conclusion

This study highlights the growing significance of PGx in pharmacy practice and underscores the professions evolving role in precision medicine. The strong recognition of PGx among pharmacists, along with their willingness to integrate it into clinical settings, demonstrates its potential to enhance medication safety and optimize therapeutic outcomes. However, several challenges persist, including educational gaps, limited accessibility to PGx testing, and the absence of standardized implementation frameworks.

To address these challenges, we recommend a multi-tiered approach. First, pharmacy education programs in Saudi Arabia and similar regions should begin revising their curricula by 2025 to incorporate comprehensive PGx content, covering both foundational genetic principles and applied clinical case studies. Second, national health authorities and pharmacy councils should initiate structured continuing education programs and certification modules to upskill practicing pharmacists. Third, collaboration among academic institutions, healthcare providers, and policymakers is necessary to develop national clinical guidelines and decision-support tools that guide the integration of PGx into routine pharmacy services.

By strengthening pharmacists’ competencies through targeted educational and institutional initiatives, the profession will be better positioned to contribute meaningfully to personalized medicine and precision-based drug therapy. Ultimately, this study lays the groundwork for future interventions that systematically integrate PGx into pharmacy practice, thereby ensuring improved patient outcomes and the advancement of individualized healthcare.

## Data Availability

Available on request.
